# PCL/PEO Polymer Membrane Prevents Biofouling in Wearable Detection Sensors

**DOI:** 10.3390/membranes13080728

**Published:** 2023-08-12

**Authors:** Roberto Delgado-Rivera, William García-Rodríguez, Luis López, Lisandro Cunci, Pedro J. Resto, Maribella Domenech

**Affiliations:** 1 Department of Chemical Engineering, University of Puerto Rico, Mayagüez Campus, Mayagüez, PR 00680, USA; roberto.delgado13@upr.edu; 2 Department of Mechanical Engineering, University of Puerto Rico, Mayagüez Campus, Mayagüez, PR 00680, USA; william.garcia10@upr.edu (W.G.-R.); pedroj.resto@upr.edu (P.J.R.); 3 Department of Chemistry, University of Puerto Rico, Río Piedras Campus, San Juan, PR 00925, USA; luis.lopez14@upr.edu (L.L.); lisandro.cunci@upr.edu (L.C.)

**Keywords:** polymer membrane, sweat wearables, biofouling prevention, biosensor

## Abstract

Technological advances in biosensing offer extraordinary opportunities to transfer technologies from a laboratory setting to clinical point-of-care applications. Recent developments in the field have focused on electrochemical and optical biosensing platforms. Unfortunately, these platforms offer relatively poor sensitivity for most of the clinically relevant targets that can be measured on the skin. In addition, the non-specific adsorption of biomolecules (biofouling) has proven to be a limiting factor compromising the longevity and performance of these detection systems. Research from our laboratory seeks to capitalize on analyte selective properties of biomaterials to achieve enhanced analyte adsorption, enrichment, and detection. Our goal is to develop a functional membrane integrated into a microfluidic sampling interface and an electrochemical sensing unit. The membrane was manufactured from a blend of Polycaprolactone (PCL) and Polyethylene oxide (PEO) through a solvent casting evaporation method. A microfluidic flow cell was developed with a micropore array that allows liquid to exit from all pores simultaneously, thereby imitating human perspiration. The electrochemical sensing unit consisted of planar gold electrodes for the monitoring of nonspecific adsorption of proteins utilizing Cyclic Voltammetry (CV) and Electrochemical Impedance Spectroscopy (EIS). The solvent casting evaporation technique proved to be an effective method to produce membranes with the desired physical properties (surface properties and wettability profile) and a highly porous and interconnected structure. Permeability data from the membrane sandwiched in the flow cell showed excellent permeation and media transfer efficiency with uniform pore activation for both active and passive sweat rates. Biofouling experiments exhibited a decrease in the extent of biofouling of electrodes protected with the PCL/PEO membrane, corroborating the capacity of our material to mitigate the effects of biofouling.

## 1. Introduction

Systems for healthcare monitoring are always evolving, mainly due to the need for enhanced biological sensing. Technological advances in wearable biosensing offer extraordinary opportunities to transfer technologies from a laboratory setting to clinical point-of-care applications [1,2,3]. Wearable devices provide a minimally invasive platform to monitor physiological events in real time by transmitting continuous data to the user’s device. Devices capable of monitoring various vital signs such as temperature, pulse and respiration rate are now part of our daily lives [1,2,3,4,5,6]. From a wearable perspective, skin-interfaced systems offer the opportunity for the monitoring of biomarkers through sweat. Sweat is a fluid rich in a multitude of molecular biomarkers such as electrolytes, hormones, amino acids, and metabolites [7,8,9,10,11]. Continuous monitoring of these biomarkers has the potential to complement traditional laboratory tests by providing immediate health data for early disease detection and personalized medicine [12].

Considerable efforts have been directed towards the elaboration of novel sensors with improved wearability profiles [2,4]. Up to this point, the majority of wearable sensor prototypes have depended on bulky systems that are mostly limited to single use, with issues regarding selectivity and sensitivity to target analytes and a lack of integrated artificial intelligence to perform multiple tasks and secure big data transfer [13]. When it comes to sweat wearables, the capability of the systems is also challenged by discrepancies in sweat rates, sweat stimulation, evaporation, sample collection, and contamination, which lead to poor performance of the sensing interface [10,11].

Sensing modes for sweat biosensors often incorporate optical measurements, enzymatic biorecognition, and electrical or electrochemical characterization [9,14]. Despite the numerous sensing options available for skin-interfaced wearable platforms, the non-specific adsorption of molecules on the sensor interface, also known as biofouling, is a common denominator limiting the longevity and performance of these detection systems. Biofouling is arguably the most significant impediment for the electrochemical performance of biosensors because it blocks the sensing interface, inhibiting analyte mass transfer, lowering electrode lifetime, and causing measurement errors [14,15]. Several studies in the field have highlighted the effect of biofouling on the performance of sweat sensors to detect trace elements [16], electrolytes [17], glucose [18], stress markers [19], and drugs [20]. Mitigation of non-specific adsorption in sweat wearables has been addressed by depositing protective layers on the surface of the electrodes [21,22,23] or by continuous replacement of the sensors [14]. Unfortunately, neither one of these alternatives has been able to fully address the issue of electrode passivation, highlighting the need for better anti-fouling methods.

Membranes offer an interesting venue since they have been used for a wide range of filtration and separation processes. Membranes are used in a variety of applications, including wastewater treatment, drinking water purification, desalination, and bioproduct separations [24,25]. A key characteristic associated with membranes is the ability to tailor their physicochemical properties to meet a particular need. Surface coatings, charge modifications, controlled surface roughness, and biomimetic strategies have played a critical role in the design and manufacturing of functional membranes.

Despite a strong track record of success associated with filtration systems incorporating membranes, biofouling of polymer matrices has been a widespread concern associated with a decrease in the response time and performance of biosensors. These limitations are often magnified for biosensors used in complex sample matrices, such as blood or sweat, hindering the diffusion of analytes and affecting real-time measurements [14]. However, great advances have been made in developing strategies to control the fouling of membranes. For technologies that couple membranes to electrochemical sensing interfaces, recent approaches use electric fields to mitigate biofouling in filtration systems [25], incorporation of silver nanoparticles in polymeric membrane ion-selective electrodes for environmental monitoring [26], and the fusion of microbial separators to protect air-breathing cathodes in fuel cells [27]. Thus, a similar approach can be implemented to develop a system for the prevention of non-specific adsorption in skin-interfaced wearable detection systems.

Despite the potential of membranes to overcome biofouling challenges, their potential for skin-contact biosensing platforms has not been well established. In the present study, we seek to capitalize on the selective properties of biomaterials [28,29,30] to achieve enhanced analyte adsorption, enrichment, and detection. Our goal is to develop a functional membrane integrated into a microfluidic sampling interface to protect a wearable electrochemical sensing unit from the effects of biofouling. The membrane was manufactured from a blend of Polycaprolactone (PCL) and Polyethylene oxide (PEO) through a solvent casting evaporation method. PCL is an hydrophobic polymer with remarkable physicochemical characteristics such as good mechanical properties, established biocompatibility, simple and easy processability, and controllable biodegradation behavior, and has been approved by the Food and Drug Administration (FDA) for biomedical applications [31]. PEO is an amphiphilic polymer with excellent biocompatibility and good solubility in water and most organic solvents. For this particular instance, PEO was chosen to increase the hydrophilicity of the cast membrane when admixed with the PCL [32] and because of its antifouling properties [33]. A microfluidic flow cell was developed to test several micropore arrays to challenge and characterize the PCL/PEO membrane in an environment imitating human perspiration. The capacity of the PCL/PEO membrane to prevent non-specific adsorption was determined using electrochemical analyses through CV and EIS measurements.

## 2. Materials and Methods

### 2.1. Polymer Solution

The polymer solution was prepared by dissolving PCL (Mw = 50,000/TP60505, Cellink, San Diego, CA, USA) and PEO (Mw = 600,000/182028, Sigma, Saint Louis, MO, USA) in dichloromethane (DCM) (270997, Sigma, Saint Louis, MO, USA) and dimethylformamide (DMF) (227056, Sigma) (3:2) at a ratio of 10% *w*/*v* and 3% *w*/*v*, respectively. The polymers were dissolved at room temperature under constant stirring until a homogeneous solution was formed.

### 2.2. Preparation of PCL/PEO Membrane

**Figure 1:**
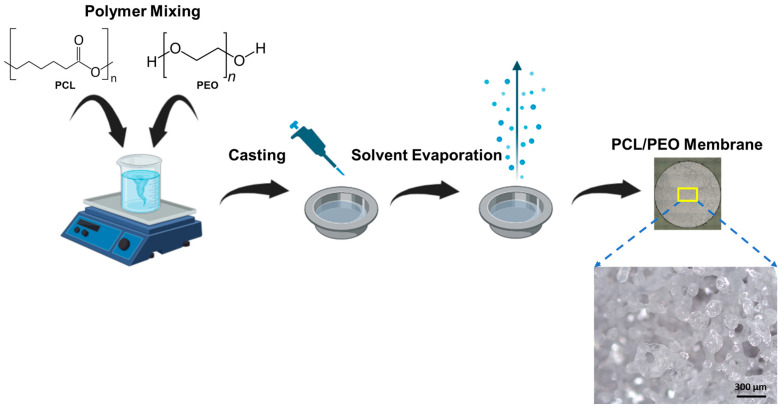
Schematic representation of the manufacturing method of the PCL/PEO membrane created with BioRender.

Membranes were prepared using a solvent casting evaporation method (Figure 1). Briefly, 2 mL of the polymer solution was deposited in an aluminum weighing boat and distributed evenly throughout the surface until an even coating was observed. The cast solution was left in a chemical hood at room temperature for roughly 24 h until the volatile solvents (DCM/DMF) evaporated, leaving a polymer film. The film was then peeled off the surface and stored in aluminum foil until used.

### 2.3. Membrane Characterization

#### 2.3.1. Optical Imaging

Bright field images of the PCL/PEO membranes were captured using a Keyence 3D laser confocal microscope (VK-X1000, Keyence, Itasca, IL, USA) to confirm the formation of the porous film once the solvent casting evaporation method was completed.

#### 2.3.2. Fourier Transform Infrared Spectroscopy (FTIR)

Functional group analysis of the PCL/PEO membrane was performed using a Perkin–Elmer Spectrum Two FTIR spectrophotometer. The PCL/PEO membrane was analyzed by peeling the membrane and mapping the surface to determine the chemical footprint of several regions within the cast material.

#### 2.3.3. Thermogravimetric Analysis (TGA)

To study the thermal stability of the PCL/PEO membrane compared to the PCL and PEO components, TGA was performed using a TGA Q50 device. Samples with weights between 10 and 20 mg were heated from 25 to 600 °C under a nitrogen atmosphere, at a heating rate of 10 °C min^−1^.

#### 2.3.4. Laser Confocal Microscopy

For confocal laser microscopy analysis, the PCL/PEO membrane was mounted on a glass microscope slide using double-sided tape. Overview images for visualization of the surface profile were taken using a Keyence 3D laser scanning confocal microscope (VK-X1000, Keyence, Itasca, IL, USA). A 3D heat map of the scanned region via image stitching, surface roughness measurements, and key surface parameters were obtained using the Keyence analysis software (v2.2.0.93).

#### 2.3.5. Scanning Electron Microscopy (SEM) and Overall Porosity

SEM was utilized to visualize the major structural elements of the PCL/PEO membranes and to determine the overall porosity of the cast material. Membrane samples were fixed to an SEM spin stub with a conductive adhesive and then coated with a thin layer of gold using a sputtering device. Images were taken as top views and cross-sections utilizing a JSM-6390 microscope (JEOL, Peabody, MA, USA).

SEM images were analyzed using the ImageJ software (v1.8.0_345) (Image J, Bethesda, MD, USA) to evaluate the overall porosity of membranes. For ImageJ analysis, the SEM images were converted to 8-bit format. Brightness and contrast adjustments were used to remove the noise in the background of the images. Automatic thresholding was then used to convert images to binary (black and white). The black areas in the converted image represent the pores, and the white areas represent the solid surface of the membrane. Five images from different castings were analyzed. The data points obtained were pooled into a single data set, where the pore distribution was represented as a frequency distribution based on size.

#### 2.3.6. Wettability

Static water contact angles were measured using an optical tensiometer (Theta Flex, Biolin Scientific, Gothenburg, Sweden). For contact angle measurements, the PCL/PEO membrane was mounted on a glass microscope slide using double-sided tape. Measurements were performed by carefully depositing a 5 µL DI water droplet on the surface of the membrane. The contact angles were measured 5 times for each sample to calculate the average value and the standard error of the measurements.

#### 2.3.7. Water Adsorption and Swelling

For water adsorption and swelling measurements, PCL/PEO membranes were punched to a circular shape. Nine samples were punched from nine different membrane castings to be evaluated as part of the experimental design. Swelling studies were performed at three different pH values (pH 5, pH 7, and pH 8) to try to cover the spectra of the pH observed in sweat. Before placing the samples in aqueous media, the dry weight of each membrane was recorded. Samples were placed in scintillation vials with 5 mL of phosphate buffered saline (PBS) at different pH values. Once the membranes were submerged in the aqueous media, they were incubated in a water bath at 37 °C under constant shaking. The weight of the wet membranes was recorded at five different timepoints: T1 = 30 min, T2 = 1 h, T3 = 2 h, T4 = 3 h, and T5 = 24 h. Before each measurement, the excess media were removed from the samples. The PBS solution was replaced after every measurement from 1 h onward. Upon the completion of the 24 h timepoint, the PBS was removed from every sample, and the membranes were allowed to dry at room temperature overnight. After the samples were dried, their respective weights were recorded once more. The swelling ratio was calculated by dividing the average wet mass of the membranes by the average dry mass of the membranes. The amount of water adsorbed was calculated by subtracting the average dry mass of the membranes from the average wet mass of the membranes. To determine if there were significant differences in water adsorption and swelling profiles between timepoints and samples from different pH conditions, a two-way ANOVA test was performed, followed by Tukey’s comparison test.

#### 2.3.8. Gravimetrical Analysis

Gravimetrical analysis of the membranes was performed by comparing the initial weight of the membranes against the weight of the membranes once the water adsorption and swelling experiments were concluded. To ensure the membranes were completely dry, they were placed in a vacuum oven at 40 °C overnight. The weight differential for each sample was calculated and pooled together to obtain an average value and the standard error of the measurements.

### 2.4. Microfluidic Flow Cell

**Figure 2:**
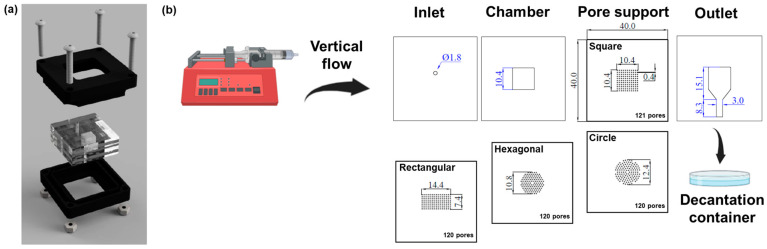
(**a**) Schematic representation of the microfluidic flow cell. (**b**) Schematic representation of the flow cell design with the different micropore array configurations: squared, circular, hexagonal, and rectangular. Scale measurements in the schematics are in mm.

The microfluidic flow cell (Figure 2) was manufactured from several cast acrylic layers each having a thickness of 3.0 mm. The area of each acrylic layer was 4 cm by 4 cm. The acrylic device was made using an EPILOG Mini CO2 60 W laser cutter (EPILOG Laser, Golden, CO, USA). The inlet and outlet ports, as well as the membrane support pores, were made by cutting all the way through the respective acrylic layer with a focused beam using the following settings: speed, 25%; power, 75%; frequency, 1000 Hz; DPI, 600. The acrylic cuts to generate individual layers were made utilizing a focused beam using the following settings: speed, 25%; power, 75%: frequency, 2400 Hz; DPI, 600. The first two and the last two layers of all the configurations were taped together using double-sided tape. The acrylic layers were sandwiched together in a plastic cassette built using a 3D printer (CR10S, Creality, Shenzhen, China).

### 2.5. Permeability and Transfer Efficiency

Permeability and transfer efficiency experiments were performed utilizing dyed DI water, a syringe pump, and the microfluidic flow cell (Figure 2b). The acrylic layers were sandwiched together in a plastic cassette. The PCL/PEO membrane was placed between the chamber and the pore support layers. Different pore support geometries were used to analyze pore activation and flow distribution through the PCL/PEO membrane. The dyed DI water was pumped through the system at a rate of 3 mL/min, simulating the sweat rate under constant exercise [34,35]. For passive sweat rate experiments, a flow rate of 10 µL/min was used [34,35]. A total volume of 250 µL was used as the experimental target. A decantation container was placed at the outlet to collect the volume transferred through the system. Volume transfer efficiency was calculated by weighing the initial volume to be dispensed and subtracting the amount of liquid collected at the outlets, using the weight of the decantation container as a baseline. The transfer efficiency experiment was performed on 5 different samples per geometry to obtain the average value and the standard error of the measurements.

As the liquid flowed through the device, a Raspberry Pi camera (ArduCam, Hong Kong, China) at a frame rate of 30 fps was used to take images with the different support geometries. Pore activation analysis was performed using the ImageJ software (v1.8.0_345). For image analysis, the images in RGB format were imported into ImageJ using the Import Image Sequence tool. The RGB channels were split into three separate images: red (R), green (G), and blue (B). For this particular instance, the B channel was the most relevant for image analysis based on the color used to dye the DI water. Pore activation was measured by counting the number of activated pores in the matrix and normalizing the data to the total number of pores available per geometry. The pore activation experiments were performed on 5 different samples per geometry to obtain the average value and the standard error of the measurements.

### 2.6. Biofouling Experiments

The prevention of non-specific adsorption was measured using electrochemical analyses. The electrochemical experiments were performed in a three-electrode cell consisting of planar gold electrodes as the working electrode, a platinum wire as the counter electrode, and an Ag|AgCl reference electrode (971-00051, Gamry Instruments, Warminster, PA, USA). All data were collected using the Gamry Instruments Framework software (v7.10.0) (Gamry Instruments, Warminster, PA, USA). Biofouling of the electrodes was promoted by incubating the working electrode with 200 µL of a concentrated solution of Fetal Bovine Serum (FBS) (29181677, Cytiva, Marlborough, MA, USA) for different time intervals (0, 15, 30, 45, and 60 min). Before every measurement was taken, the excess serum was washed away with 1 mL of DI water. The washing step was repeated three times. Once the excess serum was properly washed out of the cell, CV and EIS measurements were performed to determine the extent of the biofouling. The CV model consisted of the ability of the working electrode to measure the redox activity of a solution of 5 mM K_3_Fe(CN)_6_ (702587, Sigma) in 0.1 M KCl supporting electrolyte. The same approach was taken for the electrodes incubated with serum utilizing the PCL/PEO membrane. All measurements were compared against the baseline taken from the electrode without exposure to the serum. The anodic peak area for each timepoint was calculated by integrating the area under the curve of the CV voltammograms utilizing the Gamry Echem software (v7.10.0) (Gamry Instruments, Warminster, PA, USA). Percentage calculations were obtained by dividing the mean peak area for each timepoint against the mean area of the baseline (t = 0). For both conditions, the measurements were made in triplicate (n = 3), utilizing new electrodes each time and three different castings of the PCL/PEO membrane. Gamry Echem software (v7.10.0) was used to analyze the data and fit the equivalent circuit model to calculate the charge transfer resistance.

### 2.7. Statistical Analysis

The data represent the mean ± standard error (SE) of at least 3, 5, or 9 independent experiments as specified for each experimental condition. For comparisons of multiple means within an experimental setup, a two-way ANOVA test was performed, followed by Tukey’s comparison test. Significant changes were determined based on *p*-values: *p* < 0.05.

## 3. Results and Discussion

### 3.1. Characterization of the PCL/PEO Membrane

A standard procedure was established to manufacture a polymer membrane composed of PCL and PEO utilizing a solvent evaporation-induced phase separation casting method [36]. During the casting process, a homogeneous solution of PCL and PEO was prepared, utilizing DCM and DMF as the volatile solvents. Throughout the evaporation process, a phase separation occurs between the solvent and non-solvent parts of the mixture, resulting in a porous film. This process of forming macroporous materials using volatile solvents exposed to an atmosphere containing moisture has been formally characterized by Srinivasarao et al. [37]. Evaporation of the solvents cools the surface of the solution, initiating the nucleation and growth of moisture. When all the solvent evaporates, the film returns to room temperature, and the water droplets pack into the film. Subsequent evaporation of the water droplets is what drives the formation of the pores in the polymer film. Optical imaging of the membrane showed an opaque matrix with a porous, interconnected structure (Figure 1), corroborating the successful formation of the porous film.

The chemical structure of the PCL/PEO membrane was characterized by FTIR (Supplementary Materials Figure S1) [38,39]. FTIR analysis of several regions within the membrane showed no significant variations in the resulting spectra, hence corroborating that both polymers are present and that a uniform distribution throughout the cast membrane was obtained. In addition, the thermal stability of the PCL/PEO membrane, relative to its raw components, PCL and PEO, was studied using TGA. Thermal stability is an important factor to consider in wearable sensors since these systems are exposed to a broad range of environmental conditions, particularly for membranes in wearable systems that will be required to effectively spread and withstand any heat dissipated by the sensing unit, user, and the ambient environment [40]. As expected, PCL and PEO showed stable thermal profiles under ambient temperature and body temperature ranges (Supplementary Materials Figure S2).

**Figure 3:**
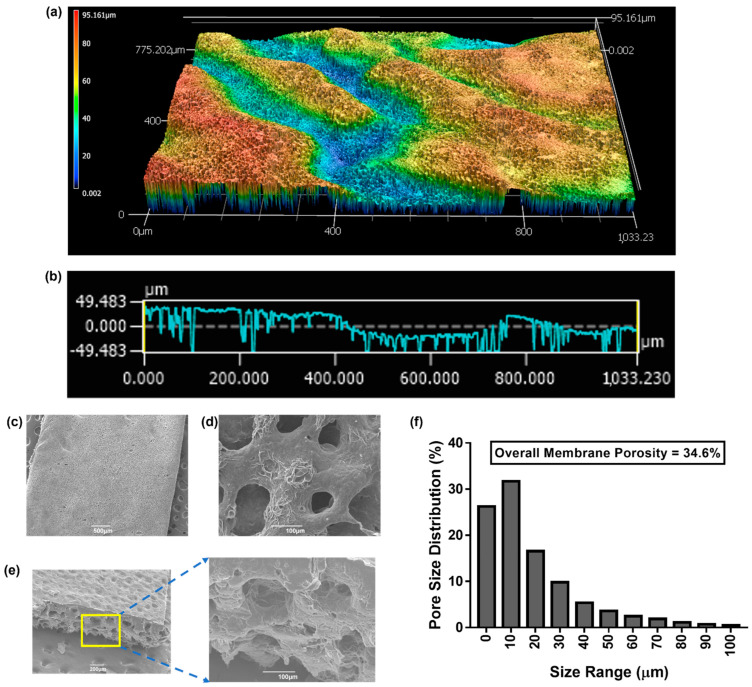
(**a**) Three-dimensional height heat map of the scanned surface. (**b**) Roughness profile of the surface scanned in panel (**a**). (**c**) SEM image of the PCL/PEO membrane. (**d**) Higher magnification SEM image of the PCL/PEO membrane illustrating the porous surface. (**e**) Cross-section SEM image of the PCL/PEO membrane. (**f**) Histogram portraying the overall porosity and pore distribution as a function of size for the PCL/PEO membrane.

Laser scanning confocal microscopy was used to perform surface analysis of the PCL/PEO membranes. Changes in focus variation were utilized to capture multiple optical images that were rendered to construct a 3D map of the surface (Figure 3a). The area scanned was roughly 1000 × 800 µm^2^. In accordance with the color code, red and blue indicate the highest and lowest points on the surface of the membrane, respectively. Surface analysis showed a rough surface with several peaks and valleys through the scanned region, depicting a maximum height of 95.2 µm. A representative 2D cross-section of the roughness profile can be seen in Figure 3b, highlighting the average difference between the peaks and valleys within the scanned area. Out of these parameters, the most relevant for potential wearable applications is the developed interfacial area ratio of 36.05. This parameter is expressed as the percentage of additional area contributed by the texture of the surface compared to the planar definition of the optical image. When it comes to the prevention of non-specific adsorption or targeted detection, this feature offers great potential to use the membranes as a filtering barrier or as a matrix support for targeted detection and enrichment.

SEM was used to obtain higher resolution images and better analyze the morphological features of the PCL/PEO membranes. Representative SEM images are shown in Figure 3c–e. The membranes formed a film that could be described as a sponge-like structure with a high degree of porosity. Cross-section images showed a packed matrix with cavities or pores throughout the entirety of the structure (Figure 3e). SEM was also utilized to determine the overall porosity of the membranes (Figure 3f). The porous structures in the membranes ranged from 10 to 100 µm, with a prevalent size distribution ranging from 5 to 30 µm. The overall surface porosity of the membranes was determined to be about 35% of the cast surface.

**Figure 4:**
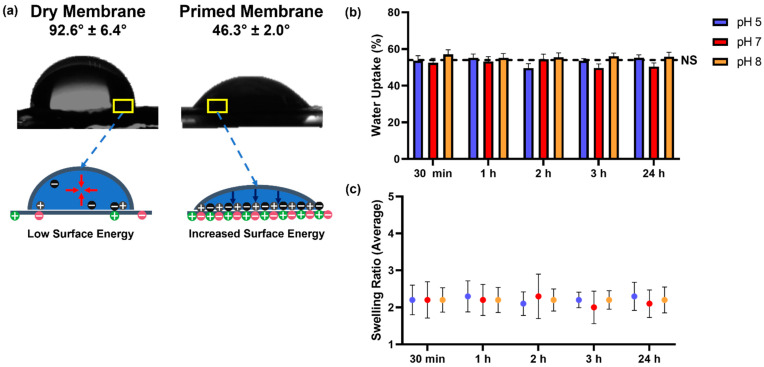
(**a**) Contact angle measurements for dry and primed PCL/PEO membranes. (**b**) Water uptake data for PCL/PEO membranes at acidic (5), neutral (7), and basic (8) pH. (**c**) Swelling profiles (normalized to initial mass of the membrane) for PCL/PEO membranes at acidic (5), neutral (7), and basic (8) pH. Contact angle data represent the mean of five independent experiments ± SE. Water uptake and swelling data represent the mean of nine independent experiments ± SE.

Contact angle measurements were taken to evaluate the surface wettability of the PCL/PEO membrane. As seen in Figure 4a, there is a big difference in the contact angle of the dry membrane (92.6°) when compared to the contact angle of the wet/primed membrane (46.3°). The polymer mixture used to manufacture the membranes is mostly PCL, a polymer that is hydrophobic in nature [31]. Therefore, at the dry stage, the surface tension of the membrane is governed by low surface energy. An interface with low surface energy is not capable of pulling the water drop out of its shape, resulting in high contact angles. It was the activation or priming of the membrane that allowed aqueous media to penetrate the matrix and increase its permeability. Activation of the matrix and the interaction of the aqueous medium with the hydrophilic part of the membrane (PEO) allowed the surface to become mostly hydrophilic with a lower contact angle. This change in surface energy is likely attributed to the addition of dipole–dipole interactions at the interface of the material. Water molecules align so that the positive end of one molecule interacts with the negative end of another molecule, pulling water towards the surface and creating a capillary effect that drives the transfer of aqueous media through the membrane [41]. To have the capillary effect as a the main conduit of our system is a remarkable feature since wearable sweat devices are required to transport aqueous media (sweat) from a limited amount of liquid volume without the help of external forces [42].

The water adsorption capacity and the swelling ratio of the PCL/PEO membrane are shown in Figure 4b,c. For water adsorption and swelling experiments, PBS at pH values ranging from acidic (pH 5) to basic (pH 8) was utilized since sweat pH can vary under different circumstances such as disease, metabolic irregularities, and stress [9,43]. Results showed that the water adsorption capacity of the membranes did not change significantly (NS) as a function of pH or the timepoint of the measurement. This finding was corroborated statistically by a two-way ANOVA test. The average aqueous media uptake was about 54% of the total mass (mass of the water adsorbed and the dry membrane) when taking into consideration all the conditions analyzed. The quantity of aqueous media captured by the membranes was normalized to the weight of the dry membranes to obtain the swelling ratio. According to the data, the membranes were adsorbing their own weight in aqueous media (swelling ratio of ~2). This was a desirable outcome due to the need for a material that can conduct aqueous media without significantly changing its physical properties in terms of size. One of the goals in the design of our system is to incorporate the PCL/PEO membrane as part of a microfluidic interface for wearable applications. Therefore, drastic changes in the swelling of the material could potentially lead to leaking of the microfluidic interface or dimensional instability of the porous structure of the membrane [44].

**Figure 5:**
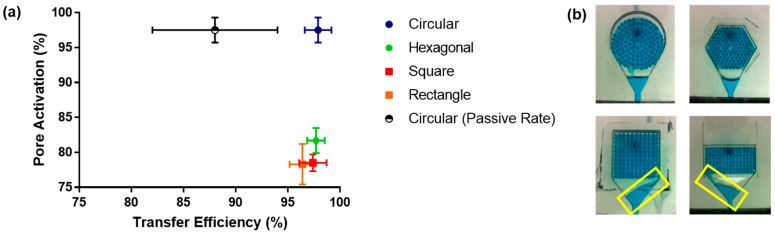
(**a**) Transfer efficiency of liquid media through the PCL/PEO membrane as a function of pore activation and membrane support geometry. (**b**) Images showing media flow through the different membrane support geometries: squared, circular, hexagonal, and rectangular. The yellow boxes highlight the fluid being pushed towards the walls of the chamber in the squared support geometries. Transfer efficiency and pore activation data represent the mean of five independent experiments ± SE.

The time it takes to measure analytes from a sweat wearable can vary depending on the device, but most of the prototypes look to provide real-time (seconds to minutes) or near-real-time (minutes to hours) measurements. Furthermore, the volume of sweat exposure in wearables can range from microliters to milliliters, depending on the sweat rate and duration of wear. Therefore, the design and formulation of any interfacial material for such an application needs to perform and be stable under this window of operation and these experimental conditions. To that end, after the water uptake and swelling experiments were concluded, we performed a gravimetrical analysis to verify the stability of the membrane. The initial composition of the PCL/PEO membrane consisted of 77% PCL and 23% PEO. By comparing the initial weight with the final weight of the membrane after 24 h of exposure to aqueous media, it was noticed that up to 20% of the initial mass was lost. Because PCL and PEO were combined without chemical crosslinking, the mass loss is most likely caused by the dissolution of PEO in the aqueous media. PEO was used to increase the hydrophilicity of the cast membrane by acting as a conduit for the capillary action required for the transport of aqueous fluids. Even after PEO was solubilized, the membrane maintained its increased wettability and uniform aqueous media transfer throughout time, as evidenced by contact angle measurements (Figure 4a) and permeability and transfer efficiency statistics (Figure 5). The loss of PEO should not substantially impact the sensor readings because, in our setup, the membrane will be wet throughout the fluid collection and transfer phase. Notably, a new formulation or the addition of a crosslinking step may be taken into consideration for wearables designed for continuous monitoring (days or weeks) to guarantee that both phases (hydrophilic and hydrophobic) stay stable, especially if there is a chance the membrane may dry out while the sensing device is in use.

### 3.2. Membrane Permeability, Transfer Efficiency and Pore Activation

To test the permeability and transfer efficiency of the PCL/PEO membrane, a microfluidic flow cell was developed (Figure 2). The flow cell was designed so that liquid exits from all pores in the micropore array simultaneously, imitating human perspiration. The testing was performed utilizing dyed DI water and a syringe pump. During the experiments, the PCL/PEO membrane was placed between the chamber and the pore support acrylic layers. Dyed DI water was pumped through the system at a rate of 3 mL/min, simulating the sweat rate under constant exercise. A total volume of 250 µL was used as the experimental target. The data suggest that, on average, no more than 9 µL of media is lost in the process of passing fluids through the flow cell with the different membrane support geometries. This translates into a transfer efficiency of aqueous media throughout the system of about 95% (Figure 5a); this is a remarkable result for the potential mass transport of limited amounts of aqueous media from sweat.

Pore activation studies utilizing different geometrical arrays showed a uniform flow through the pores of the circular geometry when compared to the hexagonal, squared, and rectangular arrays (Figure 5b). The squared geometries and the hexagonal (to some extent) arrays pushed the fluid towards the walls of the chamber, limiting the flow of media to the sides (yellow boxes) instead of utilizing the entirety of the membrane. This result was validated by digitally quantifying the percentage of pores activated throughout the transfer of the aqueous medium (Figure 5a). The circular geometry activated more than 95% of the pores in the array. Pore activation utilizing the other geometrical arrays fluctuated around 80%. The circular geometry was then utilized to test the capacity of the system to filter the aqueous media using a flow rate mimicking passive sweating conditions (10 µL/min). Pore activation within the array and the membrane remained similar to that of active sweating conditions. Regarding transfer efficiency, the data showed a diminished flow rate with greater variability, ranging from ~82% to 95% efficiency. It has been demonstrated that the mass transfer performance of filtering devices is correlated to the total volume and flow rate [45]. Hence, a decrease in transfer efficiency for passive flow rates was anticipated.

### 3.3. Biofouling Assessment

Materials with great surface area and high conductivity/sensitivity, such as metals, are excellent candidates to be used in the development of sweat biosensors [46,47]. However, metals are highly susceptible to biofouling, which can decrease the sensitivity of the sensor over time. We hypothesize that the use of the PCL/PEO membrane as a filtering barrier will decrease the rate of biofouling without posing any additional difficulty in redox transfer and increase the life of the biosensing interface. Experimentally, the biofouling prevention capacity of the PCL/PEO membrane was tested by comparing the change in the redox activity of K_3_Fe(CN)_6_ on a planar gold electrode using CV and EIS. Gold electrodes were exposed for 0, 15, 30, 45 and 60 min to a concentrated solution of FBS with and without the membrane. Under typical physiological circumstances, electrode passivation might take anywhere between a few minutes to several hours to complete. In contrast to utilizing a homogeneous protein solution, FBS can speed up the biofouling process because it is a concentrated source of a wide variety of proteins. An FBS concentration that was approximately five times higher than that utilized in cell culture applications was used to ensure quick and thorough passivation of the electrode. In this way, we ensured that we would test the ability of the PCL/PEO membrane to mitigate biofouling under what would be considered a worst-case scenario. Post FBS incubation, the electrodes were placed in a solution of 5 mM K_3_Fe(CN)_6_ to measure the change in electron transfer due to the surface blockage produced by the biofouling.

**Figure 6:**
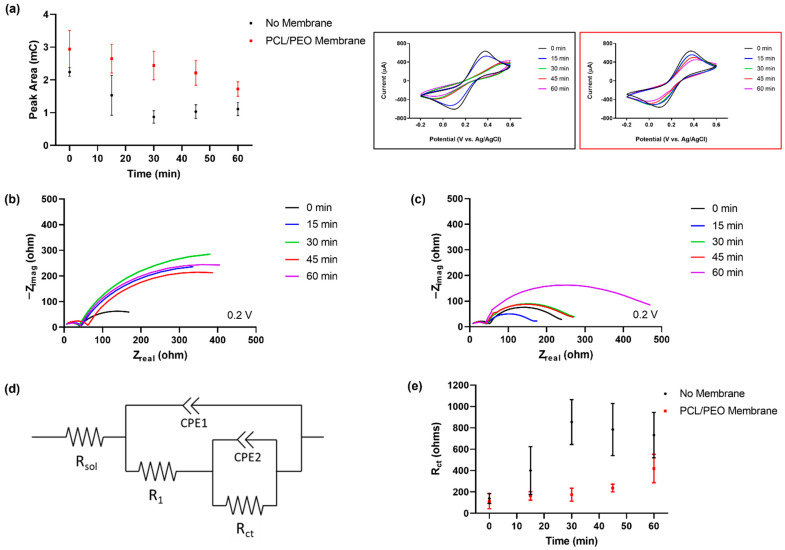
(**a**) Peak current area and cyclic voltammograms of planar gold electrodes in a solution of 5 mM K_3_Fe(CN)_6_ with a 0.1 M KCl supporting electrolyte measured after exposing the electrodes to 0, 15, 30, 45, and 60 min on a concentrated solution of FBS without (black box) and with the PCL/PEO membrane (red box). (**b**) Nyquist plot for planar gold electrode without the membrane. (**c**) Nyquist plot for planar gold electrode with the PCL/PEO membrane. (**d**) Analog electrical circuit and (**e**) charge transfer resistance over time for both electrodes. Experiments were conducted in a 5 mM K_3_Fe(CN)_6_ solution with 0.1 M KCl as the supporting electrolyte at 0.2 V vs. Ag|AgCl. Peak current area and charge transfer resistance data represent the mean of three independent experiments ± SE.

Previous studies performed by our team on metal electrodes showed that biofouling decreases the activity and reversibility of the redox reaction of Fe(CN)_6_^3+^ [48]. After exposure to FBS, the gold electrodes with and without the PCL/PEO membrane exhibited changes in reversibility, as depicted by the increase in separation and the decrease in electrical current of the redox peaks over time in Figure 6a. By comparing the peak area of the anodic current on the voltammograms from both electrodes, the membrane significantly slows the biofouling process. This effect was more evident by the 30 min mark, where the electrode without the membrane was completely passivated, whereas the membrane-protected electrode still retained about 80% of its original activity (~2 orders of magnitude difference compared to the bare electrode), as quantified by the peak current area experiments. Moreover, the membrane-covered electrodes showed a consistent change in activity and reversibility of the redox reaction between trials. On the contrary, electrodes without the protection of the PCL/PEO membrane did not have consistent changes in activity and reversibility, along with greater levels of variability. These results provided a clear indication and proof of concept of the potential of PCL/PEO-based membranes as an antibiofouling barrier for metal electrodes used in electrochemical biosensing.

EIS was also employed to study the biofouling of the electrode surface with and without the presence of the PCL/PEO membrane. Nyquist plots for the electrodes without and with the membrane are shown in Figure 6b,c, respectively. EIS data were obtained from 10 Hz to 1 MHz at 0.2 V vs. Ag|AgCl using an amplitude of 10 mV. Two reactions with different time constants are seen in each Nyquist plot. These reactions were modeled using the equivalent circuit seen in Figure 6d, a typical circuit model for electrodes with coatings. Both charge transfer resistances were calculated (R_1_ and R_ct_), with R_ct_ showing the greatest correlation with biofouling.

The charge transfer resistance (R_ct_ shown in the equivalent circuit in Figure 6d) was calculated and plotted over time to study the effects of biofouling on the surface of the electrodes. The resulting data for the change in charge transfer resistance for both electrodes are shown in Figure 6e. R_ct_ and its corresponding error values for the electrodes exposed to FBS without the PCL/PEO membrane are much higher compared to those of the membrane-protected electrodes. While all electrodes, with and without the membrane, demonstrated similar activity for the redox reaction, the differences were evident after the initial 15 min of incubation with FBS and the trend continued up to 60 min after the initial exposure to serum. At this point (60 min mark), R_ct_ values of the membrane-protected electrodes started to increase due to the biofouling of the PCL/PEO membrane. These results are in accordance with the data obtained using CV. Considering that we are promoting the biofouling process of the sensing interface with a concentrated solution of serum, the overall effect on the charge transfer resistance of the electrodes with the PCL/PEO membrane was considerably slower when compared to the bare exposed electrodes. This finding further demonstrated a decrease in the extent of biofouling of electrodes protected with the PCL/PEO membrane, corroborating the capacity of our material to mitigate the effects of non-specific adsorption.

## 4. Conclusions

In this study, we demonstrated the capability of utilizing polymer membranes as sample collection conduits with the potential of minimizing the biofouling of sensing interfaces intended for wearable detection systems. The PCL/PEO membrane presented the desired physical properties with an excellent interfacial surface ratio and ideal wettability profile (optimal hydrophobic to hydrophilic ratio). Our main target is to incorporate the PCL/PEO membrane as part of a microfluidic interface for wearable applications. Under these conditions, the main driving forces for sample collection are capillary interactions. To that end, water adsorption, swelling, and permeability experiments successfully demonstrated the capacity of the membrane to transfer aqueous media under active and passive sweating conditions with great levels of efficiency. Electrochemical data (CV and EIS) showed a significant reduction in the performance of bare electrodes that were exposed to concentrated amounts of serum when compared to the membrane-protected electrodes. These results highlight the ability of the PCL/PEO membrane to be implemented as a tool in wearable detection systems and to help retain the electrochemical performance of sensing interfaces by preventing or minimizing the non-specific adsorption of foreign molecules.

Even though significant progress has been made, challenges remain in the field of polymer membrane-based biofouling prevention. Future efforts from our group will look to optimize the design and fabrication techniques of our systems to introduce targeted detection moieties and further enhance the biofouling potential of the membranes. Moreover, testing of the PCL/PEO membrane directly in a mobile sensing interface could provide vital information to validate its performance in a relevant wearable model.

## Data Availability

The data presented in this study are available on request from the corresponding author.

## Supplementary Materials

The following supporting information can be downloaded at: https://www.mdpi.com/article/10.3390/membranes13080728/s1.
