# Supplementary material for: PCL/PEO Polymer Membrane Prevents Biofouling in Wearable Detection Sensors
Source: Membranes (Basel). 2023 Aug 12;13(8):728. doi: 10.3390/membranes13080728 (PMC10456225; doi:10.3390/membranes13080728)
Supplement: Supplementary file 1 [file membranes-13-00728-s001.zip › membranes-2518024-supplementary.pdf]

membranes

MDPI

*Supplementary Materials*

# PCL/PEO Polymer Membrane Prevents Biofouling in Wearable Detection Sensors

**Roberto Delgado-Rivera [1], William García-Rodríguez [2], Luis López [3], Lisandro Cunci [3], Pedro J. Resto [2] and Maribella Domenech [1,*]**

[1] Department of Chemical Engineering, University of Puerto Rico, Mayagüez Campus, Mayagüez 00680, Puerto Rico; roberto.delgado13@upr.edu
[2] Department of Mechanical Engineering, University of Puerto Rico, Mayagüez Campus, Mayagüez 00680, Puerto Rico; william.garcia10@upr.edu (W.G.-R.); pedroj.resto@upr.edu (P.J.R.)
[3] Department of Chemistry, University of Puerto Rico, Río Piedras Campus, San Juan 00925, Puerto Rico; luis.lopez14@upr.edu (L.L.); lisandro.cunci@upr.edu (L.C.)
* Correspondence: maribella.domenech@upr.edu; Tel.: +1809-1-(787)-832-4040

The chemical structure of the PCL/PEO membrane was characterized by FTIR. The FTIR spectrum of the PCL/PEO membrane is shown in Figure S1a. The stretching bands at ~2800 $cm^{-1}$ (black box), ~1700 $cm^{-1}$ (blue box) and ~1200 $cm^{-1}$ (purple box) correspond to the vibrations of the C-H, C=O and O-C-O bonds in PCL, respectively [38]. The vibrational bands associated with $CH_2$ groups are highlighted in orange boxes. The stretching band at ~720 $cm^{-1}$ is related to the $CH_2$ of PCL [39]. The two bands at ~1460 $cm^{-1}$ and at ~1290 $cm^{-1}$ correspond to the scissoring and twisting vibrations of $CH_2$ in PEO [39]. The band at ~1360 $cm^{-1}$ is associated with the wagging vibrations of $CH_2$ and the band at around ~950 $cm^{-1}$ is assigned to the rocking vibrations of $CH_2$, both belonging to PCL and PEO [39]. Further characterization was performed to determine the distribution of these polymers throughout the entire cast. PCL and PEO were mixed by dissolution in an organic phase. Therefore, it was imperative to understand if there was any separation between the polymers due to interfacial tension fluctuations during the solvent evaporation stage. FTIR analysis of several regions within the membrane (Figure S1b) showed no significant variations in the resulting spectra. Hence, corroborating that both polymers are present and that a uniform distribution throughout the cast membrane was obtained.

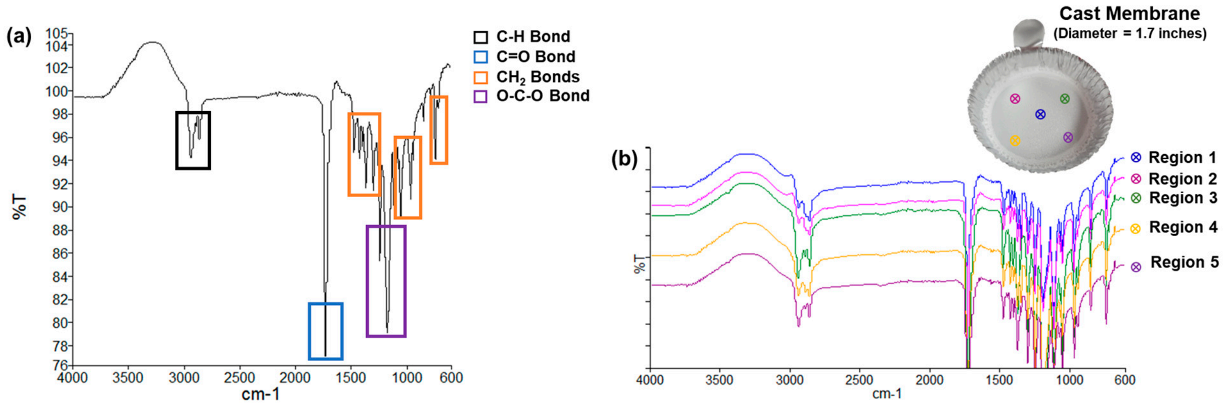


**Figure S1.** (**a**) FTIR spectra of the PCL/PEO membrane. (**b**) FTIR spectra for the PCL/PEO membrane as a function of region within the cast membrane (5 regions).

The thermal stability of the PCL/PEO membrane, relative to its raw components, PCL and PEO, was studied using TGA (Figure S2). Thermal stability is an important factor to consider since the processability of the polymers and the end use of the manufactured material may depend on it. In particular for membranes in wearable systems that will be

required to effectively spread and withstand any heat dissipated by the sensing unit, and the ambient environment [40]. PCL showed a one-step decomposition profile with a peak decomposition temperature at 442°C, as seen by the TGA and the derivative plot (DTG). PEO showed a two-step decomposition profile with a peak decomposition temperature at 422°C. As for the PCL/PEO membrane, the thermal profile was similar to that of PCL. These results were expected since the original polymer blend was 10% w/v PCL and 3% w/v PEO, respectively. The peak decomposition temperature for the PCL/PEO membrane was registered at 436°C, which falls in between the decomposition temperatures of the pure forms of PCL and PEO. In addition, at 600°C there is still a residue of ~20% of the original weight of the PCL/PEO membrane. This suggests that possible interactions between the polymers in the composite membrane are taking place in the amorphous state. Materials to be incorporated as part of wearable platforms must perform within a temperature range of 20°C to 42°C, therefore it is safe to expect that the thermal stability of the PCL/PEO membrane would not be compromised under ambient temperature and body temperature ranges.

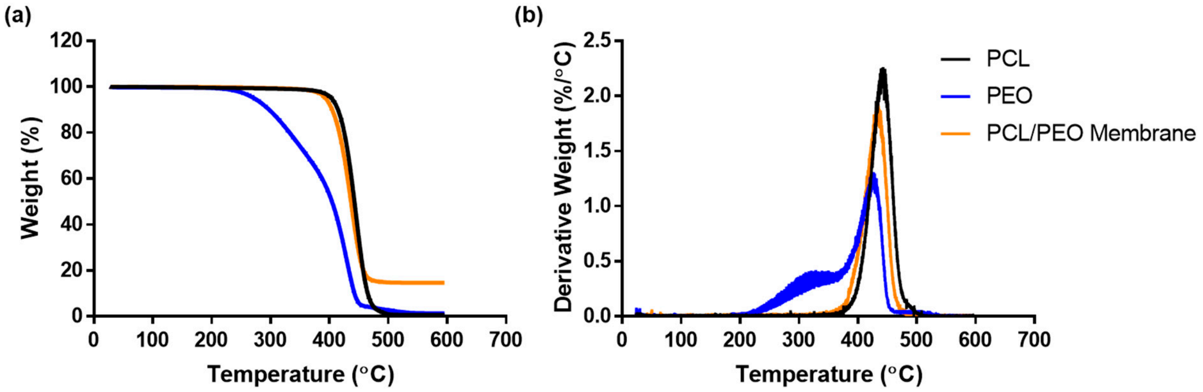


**Figure S2.** (**a**) TGA curves for PCL, PEO and PCL/PEO membrane. (**b**) Derivative weight (DTG) curves as a function of temperature.
